# Students’ perceptions on determinants of cyberbullying at a rural higher education institution in Limpopo

**DOI:** 10.1186/s12889-026-27303-6

**Published:** 2026-04-18

**Authors:** Ndzhaka Nkuna, Valeria Baloyi

**Affiliations:** Department of Psychology, University of Venda, Thohoyandou, South Africa

**Keywords:** Cyberbullying, Bullying, Social media, Online learning, Students

## Abstract

In South Africa, cyberbullying is a prevalent problem and is seen as a social and psychological concern amongst university students (Nuaimi AA. Effectiveness of Cyberbullying prevention strategies in the UAE. In ICT Analysis and Applications. 2021:731–739. Springer, Singapore). The aim of the present study was to explore students' perceptions on the determinants of cyberbullying at a rural higher institution in Limpopo province, South Africa. The study employed a qualitative approach, utilizing an exploratory research design. A sample of ten (10) students enrolled at the University of Venda (Univen) was selected through a purposive sampling method. Data was collected utilizing semi-structured interviews and analyzed through thematic content analysis. The study findings revealed that some of the determinants of cyberbullying include the need to belong, peer pressure, wanting to be famous, knowledge about victims’ personal background, and the perpetrator’s need to exert power. Conclusively, students recommend that institutions of higher learning strengthen and enforce anti-bullying policies to reduce the prevalence of this phenomenon.

## Introduction

Existing literature shows that cyberbullying is a modern-day challenge that is on the rise [36]. It is defined as any form of discomfort or harm deliberately inflicted on a specific person or group, repeatedly on social media [2, 11]. The rapid expansion of internet access and smartphone usage has created environments where harmful behaviors can occur instantly, anonymously, and on a large scale, amplifying their impact compared to traditional forms of bullying. In the digital age, where higher education institutions have adopted blended learning, students spend a significant portion of their time online, making them more vulnerable to various forms of digital harassment [14].

The university students may face cyberbullying from peers, strangers, or even individuals outside the academic environment, facilitated by the widespread use of social networking sites, messaging apps, and online discussion platforms [4, 17, 18]. As of April 2022, there were more than 5 billion internet users worldwide, accounting for 63% of the total global population. Of this total, 4.65 billion were social media users [20]. This figure is significantly higher than the number of internet users worldwide as of October 2020, which was 4.5 billion, with 3.8 billion social media users [21]. This is evidence that the digital environment is becoming an increasingly significant part of many people's lives worldwide, as internet access becomes more accessible and affordable [9, 21, 43].

Available research indicates varying levels of internet usage across different regions of the world. Approximately 80% of the population in Europe and 75% in America are online, while the Commonwealth of Independent States (CIS) records about 78%. In contrast, lower usage rates are observed in other regions: around 40% of individuals in the Arab States, Asia, and the Pacific have internet access, and only about 25% of the population in Africa is online [46]. China and India rank ahead of all other countries in terms of internet users. As of February 2022, China had more than one billion internet users, and India had approximately 658 million [20].

Research indicates that South Africa has extremely high adoption rates of mobile technology and mobile social networking usage [41, 46]. According to GSMA [13], around 79% of South Africans used mobile devices to access the internet in 2022. The digital population is also growing rapidly, with DataReportal [8] reporting approximately 43.48 million internet users in South Africa. Meanwhile, household-level data indicates that mobile internet access is the dominant mode. Generation Z in South Africa uses the internet excessively and wants to be always connected. In a study of young adults (aged 14–35) in Buffalo City, Lukose et al. [26] found that many of them spend 6 h or more daily on social media, and that FoMO (“fear of missing out”) is a significant driver of this usage [16, 38, 40]. Cilliers [6] agrees that about one‑third of university students have been victims of cyberbullying in the Eastern Cape, South Africa.

Statistics show that cyberbullying occurs among the university students [47]. Sixty-six percent (66%) of 712 public and private college and university students in Malaysia reported being cyberbullied [24]. In Pakistan, nearly 90% of respondents from the six participating universities reported cyberbullying [42]. In Israel, 57% of students had experienced cyberbullying through different kinds of media at least once or twice [39]. An exploratory study discovered that cyberbullying is common among students at a Botswana university [30]. While another study discovered that the act of deception and malice is the highest form of cyberbullying in higher learning in Kenya [33]. A recent study argues that the university environment itself can heighten the risk as students experience new freedoms, increased academic pressures, diverse social dynamics, and competitive settings. Combined with the anonymity and speed that digital platforms offer, these factors create conditions that facilitate the easy occurrence and widespread spread of cyberbullying [18].

However, Mwansa et al. [31] acknowledge the prevalence of cyberbullying at a rural-based university in the Eastern Cape, South Africa. A broader survey on learners (including university students) in Limpopo province shows that 55% had experienced some form of cyberbullying [6]. The prevalence of cyberbullying at the University of Venda, which is rural based, has been confirmed [28, 45]. However, little has been explored on the determinants of cyberbullying amongst university students. Understanding factors contributing to cyberbullying among university students is crucial, as it can influence the prevention and spread of the phenomenon. Research shows that cyberbullying is significantly associated with poor mental health outcomes among university students, including anxiety, depression, and reduced concentration [1]. Therefore, understanding the determinants of cyberbullying will inform the changes required to develop effective interventions that foster safer digital environments.

## Methodology

### Study setting

The study setting refers to the location and environment in which the research study is conducted [19]. The study was conducted at the University of Venda (commonly known as Univen). It is a South African public institution located in Thohoyandou, in the Vhembe district of Limpopo province and is the only university in the district [22, 37]. Univen was established in 1982 under the Republic of Venda government [34, 35]. It was initially created to serve the educational needs of rural and previously disadvantaged communities during the apartheid era. Over time, it has transformed into a comprehensive university committed to academic excellence, research, and community engagement. It offers a wide range of undergraduate and postgraduate programmes across several faculties, namely the Faculty of Humanities, Social Sciences and Education; Faculty of Health Sciences; Faculty of Management, Commerce and Law; and the Faculty of Science, Engineering and Agriculture. The institution places strong emphasis on fields addressing local, regional, and global development challenges, such as public health, environmental sustainability, rural development, and education.

### Research design

The current study adopted a qualitative, exploratory research design to explore students' viewpoints on the determinants of cyberbullying. The reason for choosing this design was that it enabled researchers to gain a deeper understanding of students' viewpoints on the underlying motives of cyberbullying. The new insights generated may help to inform future research and intervention strategies. Moreover, the design facilitated the exploration of this under-researched topic within the specific context of a rural institution in the Limpopo province. The use of a qualitative approach and exploratory design enabled a context-sensitive analysis that considered the social, cultural, and technological environments in which students interact. Therefore, this methodological choice not only aligns with the study’s aim of understanding students’ viewpoints but also contributed to a more comprehensive and grounded understanding of the causes of cyberbullying in a rural context.

### Population and sample

The student population at Univen is approximately 16,300 and comprises various ethnic groups, including Pedi, Swati, Tsonga, Venda, Xhosa, Shona, and Zulu. This study employed purposive sampling to select ten (10) students enrolled at Univen from all four Faculties. Purposive sampling assisted the researchers in getting information quickly and saving time because participants were targeted based on their knowledge or experience of cyberbullying. The participants in this study had to meet the following criteria: be registered students at Univen (undergraduate and postgraduate), have access to the internet, and be active on social media (e.g., WhatsApp, Facebook, Instagram, Twitter). The study excluded unregistered students at Univen and those who were inactive on social media.

### Data collection

The researchers obtained approval from the University of Venda's Research Ethics Committee (Univen REC) (Reference number FHS/23/PHYCH/12/0601) and consent from participants prior to data collection. The researcher collected data through semi-structured interviews, with the interview guide consisting of two sections: participants' demographic information and research interview questions. The interviews were conducted in English and lasted 40–60 min each. Prior to the main data collection, the interview guide was pretested with two participants who were excluded from the study sample. The researcher recorded the interviews using an audio recorder and documented field notes. The researcher booked and confirmed appointments with participants and conducted interviews at the University of Venda Library cubicles. The researcher conducted interviews with students until data saturation was achieved. Data saturation was systematically assessed throughout data collection and analysis in a sample of 10 participants.

An iterative approach was employed, whereby data collection and preliminary analysis occurred concurrently. After each interview, data was reviewed and coded to identify emerging themes and patterns related to students’ perspectives on the causes of cyberbullying. By the sixth participant, the analysis revealed that no substantially new themes or categories were emerging; instead, the data began to show repetition and convergence with previously identified findings. The researcher, however, collected data from an additional 4 participants.

### Ethical consideration

The researchers obtained approval from the Univen REC (Reference number FHS/23/PHYCH/12/0601) and verbal and written consent from participants prior to data collection. To ensure confidentiality, the researcher arranged for and requested space and privacy from the librarians for the duration of the interviews in the cubicles. Data was stored and secured in an encrypted, password-protected file on a laptop and a shared point cloud application, to which the researchers had access. Debriefing was conducted with all participants to identify any possible emotional or psychological harm for further referral to student counselling at the University of Venda. However, no harm was identified amongst the students who participated in the study.

### Data analysis

In the current study, data was analyzed using a thematic content analysis approach. Thematic analysis was employed in this study to facilitate the researcher's analysis of in-depth interview transcripts, identifying patterns and deriving themes. The researcher employed Braun and Clarke’s [5] thematic analysis process, which comprises six steps. The researcher transcribed and reviewed the collected data to identify initial patterns and codes, which were documented in a codebook. Codes were then grouped to generate and refine themes, ensuring they accurately represented the data. Each theme was defined, and illustrative examples were selected to support the analysis in relation to the research question.

### Trustworthiness of the study

#### Credibility

To ensure the credibility of this study, the researcher prioritized establishing rapport with participating students before data collection. This was achieved through initial informal interactions and clear communication about the study's purpose, ethical considerations, and the voluntary nature of participation. Furthermore, audio recordings and field notes during data collection helped prevent data loss and preserve participants’ voices. The integration of both audio data and field observations enabled methodological triangulation, thereby providing a comprehensive understanding of students’ perceptions of determinants of cyberbullying.

#### Transferability

To ensure transferability, the researchers provided clear descriptions of the Univen study setting and participants’ characteristics, allowing readers to assess the applicability of the findings to similar contexts. Furthermore, the research design was clearly outlined, including sampling procedures, data collection methods, and analytical processes. Presenting these procedures in a transparent and structured manner enhanced the potential for the study to be applied, compared, or adapted in different settings.

#### Confirmability

The researchers ensured confirmability by using audio recordings and written notes to verify that the collected data met the study objective. Direct verbatim quotes from participants were included in the findings to demonstrate that the conclusions were data driven. Additionally, a literature review was conducted to support the study’s findings with existing research, ensuring that interpretations were grounded in both the participants’ perspectives and established knowledge. A reflexive journal was also kept, recording the researcher’s assumptions and reflections, which helped to minimize personal bias during data analysis.

#### Dependability

An audit trail was maintained throughout the research process to ensure the dependability of the study. All stages of the study, including research design decisions, participant selection, data collection procedures, and data analysis processes, were thoroughly documented. The detailed reporting enables readers to evaluate the extent to which appropriate research practices have been followed. The researchers engaged peers in reviewing and discussing the data analysis process and emerging themes. This process helped to identify potential biases and to improve the consistency of the findings.

## Results

### Demographic information of participants

A total of ten (10) participants took part in the study. In terms of age, nine (09) participants were in their twenties, and one (01) participant was in her late thirties. Six (06) participants were males, and four (04) were females. In terms of education level, seven (07) participants were undergraduates, while three (03) were pursuing postgraduate studies. The participants represented all four faculties at the University of Venda. Three (03) participants came from the Faculty of Humanities, Social Sciences, and Education; two (02) from the Faculty of Health Sciences; two (02) from the Faculty of Management, Commerce, and Law; and three (03) from the Faculty of Science, Engineering, and Agriculture.

### Main results

This section focuses on students’ perceptions regarding the determinants of cyberbullying. The theme and subthemes that emerged in the current study are summarized in Table 1 below. Additionally, the subsequent sections provide a detailed discussion of the subthemes and an analysis of the consistencies and discrepancies between these findings and the existing body of literature.

**Table 1:** Theme and subthemes

Main theme	Sub-themes
3.2.1 Determinants of cyberbullying amongst university students	3.2.1.1 Early negative childhood experiences 3.2.1.2 Prejudice against specific groups 3.2.1.3 Need to exert power 3.2.1.4 Sense of belonging

#### Theme: the determinants of cyberbullying amongst university students

##### Early negative childhood experiences

The participants reported that cyberbullying stems from early negative childhood experiences of a bully, such as being abused or bullied at home, childhood trauma, lack of affection and affirmation from caregivers, and exposure to abusive behaviors in the household. These findings are supported by the following responses:

“*So, this thing, what happens is that a person who bullies people on different platforms, according to my own perspective, they are people who have suffered or been bullied mentally at home. So, they transform that behaviour from home to school or maybe to outside, or even to the world, you see*” (Participant AM).

“*There was a girl who used to be very mean and was a bully. And then we sat down this other day, and we talked about her childhood trauma and upbringing. Then she eventually opened up, saying,'You know what? I used to be told that I am ugly, which is why I behave in this certain way.' But she managed to get help in the end. Yeah, I think that was the reason that made her behave in that certain manner*” (Participant MA).

“*Problems like being abused, seeing your parents fighting, that can change your soul, growing up angry can also change you, and with that, living with an angry form, you can deviate from and break each and every rule that comes along because you don’t want to be under some rules you want to be felt, you want to be a boss, you need to be listened and known in a wrong way*” (Participant RN).

*"Let’s say the type of family in which the bully was raised. Maybe the bully is raised in a type of family where he or she is not appreciated enough. Then she goes out and she starts to body shame others because her parents do not encourage her or tell her that she is beautiful or what, because she does not get that kind of love she then tells others that they are not good enough or they are bad, then that will lead her to be a bully and then cyberbullying will take place”* (Participant VN).

##### Prejudice against specific groups

Participants in the study reported that cyberbullying can also originate from prejudice against members of specific groups due to factors such as race, culture, financial background, nationality, and family environment. The following responses support this view:

“*First of all, what can be the problem is a different culture, different race, different point of view, different backgrounds, you see, different expectations and how we raise our points that’s what going to cause the bullying because we don’t see things the same way we’re not coming from the same culture, the dressing attire is not the same, you see, what we eat is not the same, you eat Mopani worms, I eat something else, some people will mock you for that. We are coming from a poor background, coming from a poor background, I eat certain things which, for you, they seem to be lower-grade food, so that’s where bullying comes in. From different places, countries, and provinces, you see that there are even people who come from rural and urban areas. Therefore, there are many factors that can contribute to such issues at school” (*Participant AM).

“*When you have an institution that brings together people you know as we are here now like in this place, no matter how it may look like a local school we still have people from different cultures, from different homes, from different environment so when they come together in an environment like this, you are expected to find different characters because we are coming from different background*s *and bullies take advantage’’* (Participant FJ).

“*You have your flaws, and I have mine. Nobody is perfect. Go back to people’s families, you’ll see; you’ll understand where they come from, to be honest with you. If you know that person personally, you will know that the person might have anger issues that aren’t resolved. So, whatever they do, they don’t care about the second person, and therefore, they will do anything to feel better’’ (*Participant MA).

“*We have factors like environmental factors, like maybe that person is not used to that environment. Let’s say I am from Mozambique and I'm coming to Limpopo now. Since I am not accustomed to the Limpopo lifestyle, I will struggle to make friends due to my background. People might take me differently from those friends who were born in Limpopo. So, then I may develop isolation, maybe I will isolate myself from people because they don’t take me as a normal person. So, during my spare time, it’s likely that I will maybe be focusing more on Facebook, WhatsApp, and so on, and that is where the problems develop*” (Participant VN).

“*We all know that we are all different. We can’t be the same, you know, you may lack in this, and I don’t lack in that. And what I lack in, you may lack in it. We don’t come from the same financial background, you know. Some are disadvantaged, you know, and it’s not their choice to be raised and to grow up in a poor family or something like that. Yeah, so you just have to tell yourself that we are all the same, even though we are not the same*” (Participant GV).

##### Need to exert power

Participants in the study reported the need to exert power on victims as one of the causes of cyberbullying. This finding was supported by the following responses:

*“In other platforms, just to embarrass someone, or maybe if you feel like you are overpowering the person through authority, or maybe by age, that’s what mostly happens"* (Participant AM).

“*Most of the time, perpetrators are those people who get more power using these social media accounts just for fun and to make sure that other people don’t become higher than them. They want you to stay below them and have low self-esteem because they feel powerful whenever they own you. I think that’s the reason why they do it*” (Participant RN).

*“Bullies love power and feel good when they say nasty things about others, when they have audiences that love the behaviour, they get worse’’ (*Participant PM).

##### Sense of belonging

Participants in the study indicated that the need to belong, as shown in Table 1, which is ignited by peer pressure and wanting to be famous, contributes to the occurrence of cyberbullying. This was supported by the following responses:

“*But then there are people who are not bullies at all. But they are joining that thing to fit in with a certain group. So, in order to fit in a certain group of bullies, you also have to bully people so that they can fit in* (Participant AM).

“*So, you need to find that problem and ask questions like, for a person to bully other people in the community, what is the reason? Mostly, it can be peer pressure”*, (Participant RN).

*“I think it is for fame. They want to be famous using other people’s names’’* (Participant PM).

“*I think it’s because some people will do anything to feel good about themselves while crushing the ego of another person, so it actually shows that they don’t even care what happens to you*” (Participant MA).

## Discussions

The findings of the current study indicate that the early negative childhood experiences of the perpetrator play a significant role in students’ engagement in cyberbullying, particularly experiences of abuse, neglect, and exposure to conflict within the home environment (see Table 1). Participants reported that perpetrators of cyberbullying often come from households characterized by hostility and frequent parental conflict, which may contribute to the normalization of aggressive behaviors and their subsequent expression in online interactions with peers [12, 49]. The aforesaid results may be applicable to perpetrators of cyberbullying at Univen, where a substantial number of students come from underprivileged backgrounds. Additionally, domestic violence incidents are still rife in rural areas of Limpopo province [27], and conditions that have been associated with exposure to familial aggression. Such exposure may contribute to the development of aggressive or maladaptive coping behaviors among some students, which can manifest in the perpetration of cyberbullying within the university context.

The current study agrees that students who have experienced or witnessed abuse and trauma during childhood tend to internalize anger and aggression. Consequently, students who are exposed to abuse or recurrent parental conflict during childhood are adversely affected by such experiences, which may contribute to defiance toward rules and authority and, in turn, the adoption of cyberbullying behaviors as a means of expression [15, 25]. Therefore, context-sensitive interventions that acknowledge the influence of students’ familial and socio-economic backgrounds are needed. Promoting the utilization of counseling services among students is essential, particularly in rural contexts where mental health challenges are often compounded by stigma. The implementation of peer support programs and awareness campaigns that foster emotional regulation and encourage responsible online behaviors is critical for enhancing students’ psychological well-being and mitigating harmful digital interactions. Similarly, this study shows that students raised in families where emotional support and affection were lacking are more likely to direct unresolved emotional distress toward others through online harassment, rather than adopting constructive coping strategies. These findings are consistent with General Strain Theory [51], which posits that exposure to persistent stressors and adverse life experiences generates frustration and anger that increase the likelihood of deviant behaviors, including cyberbullying, as coping mechanisms.

Furthermore, the findings of the current study indicate that prejudice toward specific social groups based on factors such as race, culture, socioeconomic status, nationality, and family background contributes significantly to students’ experiences of cyberbullying. This finding is supported by Cummings [7], who identified culture, age, socioeconomic status, and other social classification variables as key factors underlying cyberbullying behaviors. Similarly, Zampieri et al. [50] reported that existing research has predominantly focused on characteristics such as social class, gender, body weight, sexual orientation, race, and physical appearance as common bases for victimization. West [48] further noted that cyberbullies themselves acknowledged targeting victims based on a range of personal characteristics, including appearance, gender, intelligence, religion, ethnicity, disability, and sexual orientation.

However, this study suggests that cyberbullying is often rooted in social hierarchies and power imbalances, where perceived differences are exploited to marginalize and harm others. In addition, students’ diverse home backgrounds contribute to variations in values and attitudes, with those from affluent socioeconomic contexts sometimes displaying prejudicial attitudes toward peers from lower socioeconomic backgrounds [23]. Although the findings indicate that external and contextual factors may influence cyberbullying behavior, Choice Theory (CT) offers a contrasting perspective by emphasizing individual agency and personal responsibility. According to CT, individuals have power and control over their own behavior, but limited control over others' behaviors and emotions. From this theoretical standpoint, prejudice related to race, culture, socioeconomic status, nationality, or family environment cannot be regarded as the primary driving force behind perpetrators’ engagement in cyberbullying. Rather, cyberbullying is viewed as a deliberate behavioral choice, shaped by an individual’s internal motivations and decision-making processes. Perhaps this would explain the reason the current study found that the desire to exert power plays a central role in cyberbullying perpetration.

Participants indicated that perpetrators often engage in cyberbullying to dominate or overpower their victims, frequently by exploiting social media platforms to publicly expose or humiliate others, thereby asserting control and superiority. Supporting this finding, Myers and Cowie [32] reported that students who engage in cyberbullying commonly cite provocation, entertainment, and the pursuit of power and status as motivating factors. Similarly, West [48] found that perpetrators acknowledged cyberbullying peers out of anger and for amusement. Meanwhile, Menesini et al. [29] demonstrated that cyberbullying behaviors can be predicted by self-enhancement motives, whereby students who prioritize self-promotion are more likely to seek power, dominance, social recognition, and success through harmful online behaviors. In agreement, the current study found that some perpetrators engage in cyberbullying to gain acceptance within specific peer groups, to impress others, or to achieve fame and popularity at the expense of their victims. Supporting these findings, Tanrikulu [44] reported that cyberbullying behaviors tend to increase when individuals’ needs for belonging are insufficiently met. A sense of belonging is widely recognized as a fundamental human need, essential for psychological well-being and healthy social functioning [10]. Correspondingly, participants in the current study indicated that students are often inclined to mimic their peers' behaviors, both positive and negative, to secure social acceptance. Beyens et al. [3] similarly noted that individuals’ use of social media is strongly motivated by the desire to belong and maintain connections within peer networks. As a result, some students may resort to cyberbullying without fully considering the harmful consequences for victims.

From a CT perspective, cyberbullying represents a maladaptive strategy that emerges when individuals are unable to fulfill their need for belonging through constructive and socially acceptable means [44]. Although such behavior is considered deviant, CT emphasizes that perpetrators remain responsible for their actions, as their behavior is ultimately a choice. In this context, cyberbullying provides perpetrators with a distorted sense of connection, recognition, and acceptance from like-minded peers within their social circles, thereby reinforcing the behavior despite its negative impact on others. Therefore, rural institutions of higher education should prioritize measures that promote and enforce safe digital engagement, ensuring that clear policies and accountability mechanisms are in place to hold perpetrators of cyberbullying accountable for their actions. Furthermore, targeted support for students from vulnerable family backgrounds, along with staff training to identify and respond to signs of distress and online aggression, may contribute to reducing cyberbullying and fostering safer digital environments within rural higher education contexts.

## Limitations and recommendations of the study

The participants in the study were all selected from Univen in Limpopo province, South Africa. Therefore, the participants' viewpoints cannot be generalised to other university students, as the contexts and backgrounds may differ. The study further relied on a purposive sample of only ten participants. While this allowed for in-depth exploration, the small sample size restricted the ability to draw broad conclusions or to identify patterns of cyberbullying that may exist in larger populations. Additionally, the study focused primarily on students' perceptions, without examining other influential factors such as personality traits, socio-economic status, or digital literacy, which could have provided a more comprehensive understanding of cyberbullying.

Data was collected through interviews, which depended on participants’ honesty and ability to recall experiences accurately. Social desirability bias may have led some participants to underreport or overreport certain behaviors related to the phenomenon. The current study recommends that higher education institution could implement educational programs that promote diversity awareness, cultural sensitivity, and social justice to help challenge stereotypes and reduce prejudicial attitudes among students. Furthermore, creating safe reporting mechanisms and support systems for marginalized students can mitigate the harmful effects of cyberbullying. Thus, by fostering inclusive campus cultures that value diversity and equity, higher education institutions can reduce the prevalence of cyberbullying rooted in social prejudice and promote respectful online engagement among students. Future research can explore the phenomenon using various research approaches and designs, as well as larger sample sizes. Future researchers can also explore context-specific strategies that students can utilise to promote safe digital engagement in addressing the cyberbullying phenomenon. Moreover, exploring factors associated with cyberbullying from victims’ viewpoints can offer a more comprehensive understanding of its impact.

## Data Availability

The data supporting the findings of this study are not publicly available due to the need to protect participants' privacy, but are available from the corresponding author upon reasonable request.

## Abbreviations

CIS: Commonwealth of Independent States
FOMO: Fear of Missing Out
Univen: University of Venda
Univen REC: University of Venda Research Ethics Committee
CT: Choice Theory
